# Enhancing neuropsychological assessment clinical pathways in Parkinson's disease through the use of technology

**DOI:** 10.3389/fdgth.2025.1681221

**Published:** 2025-10-23

**Authors:** Elton H. Lobo, Peter Worthy, Deborah Brooks, Kirstine Shrubsole, Dana Pourzinal, Deepa Sriram, Rachel Fels, Ji Hyun Yang, Jacki Liddle, Leander K. Mitchell, Nadeeka N. Dissanayaka

**Affiliations:** ^1^ UQ Centre for Clinical Research, Faculty of Medicine, The University of Queensland, Brisbane, QLD, Australia; ^2^ School of Electrical Engineering and Computer Science, The University of Queensland, Brisbane, QLD, Australia; ^3^ School of Health and Rehabilitation Sciences, The University of Queensland, Brisbane, QLD, Australia; ^4^ Queensland Aphasia Research Centre, The University of Queensland, Herston, QLD, Australia; ^5^ Department of Occupational Therapy, Princess Alexandra Hospital, Brisbane, QLD, Australia; ^6^ School of Psychology, The University of Queensland, Brisbane, QLD, Australia

**Keywords:** cognition, journey map, neuropsychological assessment, Parkinson's disease, technology

## Abstract

**Background:**

Routine administration of neuropsychological assessments to evaluate cognitive decline in Parkinson's disease (PD) may not be feasible in current clinical services. This is due to lengthy administration time, lack of specialised neuropsychologists and other limitations in resources. While technology integration could improve efficiency, understanding the existing assessment journey is crucial for successful implementation in clinical services. This preliminary study from the PDCogniCare project aims to explore current practice in neuropsychological assessments for people with PD and identify opportunities for technological integration.

**Methods:**

A qualitative study using semi-structured interviews was conducted with 15 clinical experts across two public health services in Australia. Data were analysed using inductive coding and journey mapping approaches to develop a comprehensive map of neuropsychological assessment journey.

**Results:**

Analysis revealed a four-phase assessment journey: initiation, brief cognitive screening, detailed neuropsychological assessment, and feedback, with distinct variations between clinical pathways. Key challenges included long waiting times, assessment duration, complex reporting, and limited awareness of cognitive assessments. While technology integration could begin to address some of these challenges through streamlined processes and improved access, barriers such as system integration, user adoption, and assessment methodology constraints require consideration.

**Conclusion:**

This study revealed the complexity of neuropsychological assessment pathways and identified potential areas for technological enhancement. Future research from the PDCogniCare project will aim to address these areas by employing appropriate methodologies and theoretical models to guide the design and development of technologies for neuropsychological assessments in PD.

## Introduction

People living with Parkinson's disease (PD) are at elevated risk of developing dementia (1). This risk increases as the disease progresses (2). Neuropsychological assessment is a standard approach for evaluating cognitive decline associated with the onset of dementia in PD (2–4). These assessments provide standardised metrics for examining brain-behaviour relationships, evaluating cognitive deficits, and identifying patterns in cognition linked to brain disorders (5). While neuropsychological assessments are useful, they take several hours to conduct and are not always feasible within routine clinical practice (6). These assessments are also impacted by costs and limited access to neuropsychology services (7). Consequently, shorter cognitive assessments, including brief instruments such as the Mini-Mental State Examination (MMSE) or the Montreal Cognitive Assessment (MoCA), are often used as an initial screening step (8). However, there is limited clarity regarding the selection and timing of assessments, as well as the standardisation of clinical processes (9–11). Further research is therefore needed to understand these diverse clinical pathways for assessing cognitive impairment and dementia risk in people with PD. This need is also driven by people with PD who require prioritised psychological research on cognitive functioning to understand how the condition influences their cognitive abilities and to obtain practical information for managing cognitive symptoms (12).

Clinical pathways, grounded in evidence and clinical guidelines, have demonstrated effectiveness in healthcare delivery (13). This effectiveness could be enhanced through the integration of health information technology (13). Health information technology, particularly telehealth, has shown significant potential in improving access to healthcare services regardless of geographic location, while also reducing healthcare costs (14, 15). Additionally, it showed good reliability and agreement compared to face-to-face assessments. For instance, Hernandez et al. (16) found good reliability (ICC = 0.80–0.82) between face-to-face and remote cognitive testing in older adults, though remote scores were significantly higher for Abbreviated Mental Test (AMT) and Chinese Mini-Mental State Examination (mCMMSE). Another study by Zadik et al. (17) found excellent agreement (ICC = 0.89) in total MOCA score between face-to-face and via videoconference using a mobile phone. Beyond reliability and agreement, the study by Bălăeţ et al. (18) discussed the various benefits of technology-based cognitive assessments over traditional pen-and-paper scales. These benefits included reducing administrative costs and travel burdens while enabling repeated testing, gamification, and large-scale longitudinal monitoring from home (18). Furthermore, such technologies provide automated scoring, streamlined data management, and detailed performance modelling that can isolate specific cognitive processing components such as visuomotor slowing (18). This potential offers advantages in neuropsychological assessment processes, where there is a growing interest (19, 20). PDCogniCare is an Australian project that aims to address this need by improving the delivery of neuropsychological services for people with PD through technology. Understanding how novel technologies integrate within existing clinical pathways is crucial when implementing new solutions (21), and hence a key tenant in the PDCogniCare project.

In the past, journey mapping approaches have shown significant potential in identifying and analysing clinical pathways (22). Journey mapping looks to create a visual timeline that illustrates the multidimensional relationship between the individual and the health service (23). This visual representation helps identify gaps in health service, allowing for improvements to the overall patient experience and health outcomes (24). Journey mapping in medical research remains an emerging field (23), with a notable lack of research specifically addressing neuropsychological assessments and opportunities for technological integration. This study aims to explore how journey mapping applies to understand clinical pathways in neuropsychological assessments in PD and identify potential areas where technology could be integrated to improve overall clinical workflows.

## Methods

### Study design

The study used a qualitative descriptive design based on semi-structured interviews. The semi-structured qualitative interviews were conducted with clinical experts from two public outpatient clinical services. Interviews aimed to understand the current practices in conducting neuropsychological assessments and potential for technology integration. A journey map of their experiences in conducting and/or utilising results of neuropsychological assessments in their roles within public health services in Queensland, Australia was created. The study was approved as part of the PDCogniCare project by the Metro North Health Human Research Ethics Committee (Project ID: 100098), and the University of Queensland Human Research Ethics Committee (Project Number: 2023/HE002029).

### Participant recruitment

Clinical experts, including neurologists, geriatricians, neuropsychologists, movement disorders nurses, psychiatrists, and allied health professionals, were recruited from each public health service. Clinical experts were purposefully selected based on their experience working with people with PD. Purposeful sampling was employed to select participants who could provide detailed and insightful information on the phenomenon being studied (25). In this case, the phenomenon focused on experience with conducting or referring for neuropsychological assessments in PD. Participants were recruited through a snowballing approach, including personal contacts and contacts of research participants.

### Data collection

Semi-structured interviews were conducted via video conferencing platforms such as Zoom and Microsoft Teams by five researchers (DB, JY, KS, LM and PW). An interview guide was developed based on two frameworks of the Theoretical Domains Framework (26) and the Consolidated Framework for Implementation Research (27) to determine potential opportunities for the implementation of a technology within the clinical pathways. It covered topics such as (i) standard practices for conducting cognitive assessments, (ii) needs related to routine cognitive assessments, and (iii) the potential of technology to address existing limitations. Each interview took approximately 60 min and was audio and/or video recorded with the participants' consent, and transcribed verbatim.

### Data analysis

Transcripts were transferred to NVivo 12 software for analysis. After familiarisation, qualitative content analysis was performed (28). The coded text was combined by identifying key similarities and differences in the journey and altering the pathways to ensure comprehensiveness. This was conducted in consultation with the wider author group to create a preliminary journey map. This map helped to understand the structure of the overall assessment pathway. It also highlighted potential areas of weakness and informed approaches for integrating technology. These insights contributed to the development of a comprehensive assessment journey.

Initially, eight transcripts from clinician stakeholders were extracted and coded. These codes were mapped to the pilot map, resulting in eight distinct journeys. These journeys were grouped by public health service. The journey maps within each group were compared to identify similarities and differences, which were merged to form a preliminary assessment map. This map was expanded by incorporating seven additional clinician interview transcripts. After multiple iterations, two pathways were identified, one for each public health service. The pathways were simplified and condensed into a single comprehensive assessment journey representing neuropsychological assessments at a public health setting in Queensland, Australia. Further, this map outlined potential approaches for technology integration.

## Results

In total 15 clinical experts (8 men and 7 women), aged between 30 and 69, were interviewed. Clinical experts included 5 neuropsychologists, 4 consultant neurologists, 2 consultant psychiatrists, 2 movement disorder nurses, a geriatrician, and 1 speech pathologist. Their combined experience ranged from 1 to 29 years in their current roles. One participant did not disclose their age group, and another did not provide their years of experience. The demographic data of each participant is presented in Table 1.

**Table 1:** Participant demographics.

Characteristics	Number of participants, *n* (%)
Current role	
Neuropsychologist	5 (33.3%)
Neurologist	4 (26.7%)
Psychiatrist	2 (13.3%)
Movement disorder nurse	2 13.3%)
Geriatrician	1 (6.7%)
Speech pathologist	1 (6.7%)
Age group	
30–39	4 (26.7%)
40–49	4 (26.7%)
50–59	5 (33.3%)
60–69	1 (6.7%)
Not described	1 (6.7%)
Years of experience	
1–4	4 (26.7%)
5–9	5 (33.3%)
15–19	1 (6.7%)
20–24	3 (20.0%)
25–29	1 (6.7%)
Not described	1 (6.7%)
Service	
Public health service 1	7 (46.7%)
Public health service 2	8 (53.3%)

### Neuropsychological assessment journey

**Figure 1:**
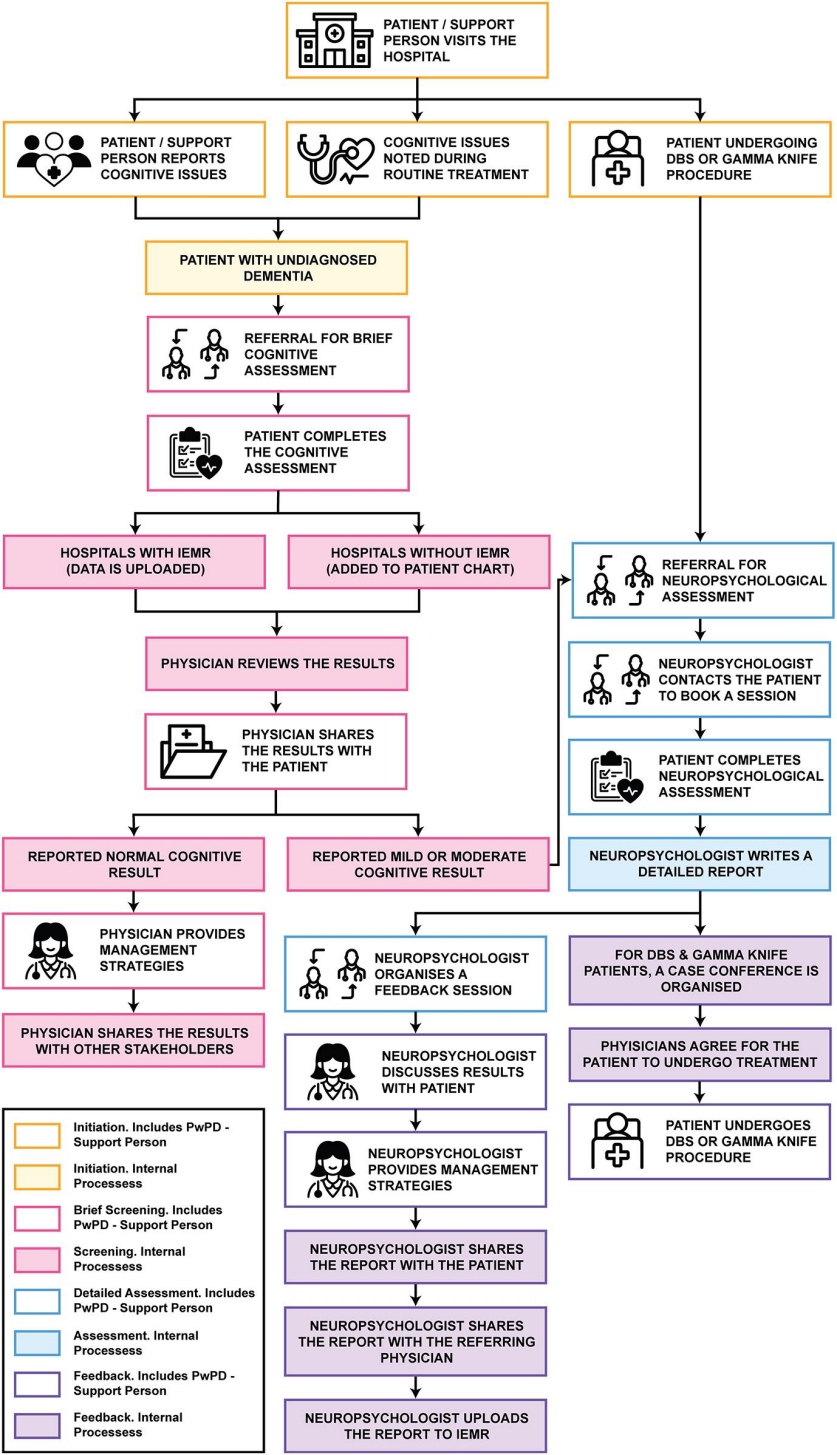
Assessment journey illustrating the typical stages of neuropsychological assessment. Icons reproduced from Noun Project.

The neuropsychological assessment process was categorised into four distinct phases: initiation, brief cognitive screening, detailed neuropsychological assessment and feedback. Each phase involves the participation of people with PD, their support person, and either a nurse or physician, as illustrated in Figure 1. It is important to acknowledge the existence of two distinct clinical pathways (*CP1* and *CP2*) that were constructed from data from two different public health service, each operating with their own established procedures.

#### Phase 1. Initiation

The initiation phase marks the point at which the need for cognitive assessment is identified. This can occur when cognitive issues are reported by the person with PD or their support person, or when cognitive concerns are detected during routine assessments. Additionally, this phase may be triggered when the person with PD is being considered for deep brain stimulation (DBS) or gamma knife procedures. For people with PD with undiagnosed dementia that have reported cognitive issues or if cognitive issues are identified during routine assessments, referrals for cognitive assessment are made by the neurologist (*CP1* and *CP2*) or by the geriatrician (*CP2*). However, for PD-Dementia patients, they would undergo discussions with the neurologists (*CP1*).

For individuals being considered for DBS or gamma knife procedures, referral to a neuropsychologist for a comprehensive neuropsychological assessment is standard across both clinical pathways. In *CP1*, this referral is facilitated by the neurologist, while in CP2, either a neurologist or a geriatrician may initiate the referral process.

#### Phase 2. Brief cognitive screening

The brief cognitive screening phase involves conducting an assessment to confirm the presence of cognitive impairment in the person living with PD. In both clinical pathways, the individual undergoes a brief cognitive assessment, which may be administered by a trained neurologist (*CP1* and *CP2*), geriatrician (*CP2*), psychiatrist (*CP1* and *CP2*), registrar (*CP1* and *CP2*), or nurse (*CP2*). While these assessments are generally performed within an outpatient setting in both pathways, *CP2* also included the provision for trained nurses to conduct cognitive assessments at the individual's home. The data collected from these assessments in both *CP1 and CP2* were uploaded to the integrated electronic medical record (iEMR) where available, or alternatively, recorded in the patient's chart (*CP1*).

Once added to the iEMR or patient chart, the primary physician, either a neurologist (*CP1 and CP2*) or a geriatrician (*CP2*), reviews the results of the brief cognitive assessment. In certain cases, particularly in *CP2*, the primary physician may refer the person with PD to a speech pathologist if their cognitive assessment results are borderline, to evaluate the impact of communication on cognition. The speech pathologist's findings are then verbally communicated to the primary physician. The physician subsequently shares the results with the person living with PD, their support person, and, in *CP1*, their general practitioner (GP). For individuals with normal cognitive finding, the primary physician provides preventive management strategies and symptom control. However, in cases of mild or moderate cognitive functioning, a referral is sometimes made to a neuropsychologist for a comprehensive neuropsychological assessment. This referral typically includes the referral letter, patient notes and brief cognitive assessment results, which in *CP1* are sent directly by the primary physician to the neuropsychologist, while in *CP2*, the nurse facilitates the referral process.

#### Phase 3. Detailed neuropsychological assessment

While both *CP1* and *CP2* followed a similar process during the in-depth neuropsychological assessment phase, *CP1* includes an additional preliminary step in which the neuropsychologist contacts the person with PD for a 30-minute call to review their case and schedule the appointment. During the visit to the neuropsychologist, the person with PD undergoes a comprehensive battery of assessments, conducted over a 2–5-h appointment. Upon completion of the assessment, the neuropsychologist schedules a feedback session with the person with PD and their support person to discuss the findings of the assessment.

#### Phase 4. Feedback

Following the detailed neuropsychological assessment, the neuropsychologist reviews the results and prepares a detailed report. This report is scanned and uploaded into the iEMR and is subsequently shared with the referring physician or nurse. For individuals following the DBS or gamma knife procedure track, a case conference is convened in *CP2*, involving relevant physicians and nurses, to assess eligibility for the procedure. If deemed eligible, the primary physician proceeds with the referral for the procedure. For persons with PD on the alternate journey, the results are communicated during the feedback session, and appropriate management strategies are provided by the neuropsychologist. Moreover, in *CP2*, the referring physician or nurse typically shares the results with the person with PD's general practitioner to facilitate ongoing support within the community.

### Opportunities for technology integration

During the neuropsychological assessment process for people with PD, participants identified several technological opportunities to enhance clinical care delivery (Table 2). Among these opportunities, technology-enabled training support emerged as a promising solution towards delivering specialised training across multiple roles in the assessment pathway. For instance, at the brief cognitive screening phase, nurse navigators in *CP2* conducted screenings but required additional training for this role. Neurologists needed enhanced knowledge regarding referral pathways after receiving cognitive assessment results. At the detailed neuropsychological assessment and feedback phases, neuropsychologists across both clinical pathways required training in PD-specific practices.

**Table 2:** Opportunities, benefits and barriers to technology integration.

Theme	Quote
Technology opportunities	
Training and recommendations	▪ “*You know, unless you are completely new to the, you know, clinical assessment with people with Parkinson's, then you [Clinician] might benefit from some training modules embedded in the program*” -Neuropsychologist ▪ “*you know listing recommendations for treatment as well from the psychologist and that*” -Neurologist ▪ “*if it's a say something that's being given on a tablet or something. then of course, it would need to be a little bit of training to work out*” -Neurologist
Access to summary reports	▪ “*to be able to access like a report – a summary report of like the implications, um, I think would – would have to be a must*” -Neurologist
Visualisation of reports	▪ “*The results were, they [Neurologist] thought everything we put in are important and they want to see all the percentiles. And we're like, “Well, then don't say you don't read — you haven't read it because it's long*” -Neuropsychologist
Centralised platform	▪ “*I guess some of the referral forms that we use currently, um, they're a bit all over the place I find, and it'd be nice if – look, I guess the forms that I found really easy to fill out are the ones where, um, they have the information that they're needing from me, either a dropdown box, or a space that I can fill it*” -Nurse Navigator ▪ “*Um, in terms of patient records, tracking their hospital stays, looking at all their notes, um, and things like that. Um, but in saying that, um, I guess, oh, with – is that a good term – ah, I guess, when we write clinical letters, ah, we either physically write them, or we dictate them*” -Nurse Navigator
Benefits to technology integration	
Improve clinical processes	▪ “*What's — you know, what's good for the patient in it, you know, is it automatic reminders of referrals? Is it, you know, triggers referrals for more comprehensive assessments when they get this [brief cognitive assessment] score*” -Neuropsychologist ▪ “*I think if I knew that uh patients would be seen in a timely fashion and the results are, you know, easily to easy to access*” -Neurologist ▪ “*a flagging once an – an assessment's done*” -Speech Pathologist
Reduce missed information for referrals	▪ “*I think this, you know, times always affect this, so the the less amount of information that would have a doctor has to import to refer the the better. Obviously you don't wanna miss any relevant referral details, but yeah.*” -Neurologist
Benefit for remote and regional areas	▪ “*May also be very useful for non-metro areas. They have good services but fewer resources and access to cognitive assessments*” -Neurologist
Provides timely access to data	▪ “*think uh, having results that are easily accessible and come back in a timely fashion*” -Neurologist ▪ “*its [cognitive assessment results] going to be more helpful for the family if the patients are happy for them to view it*” -Neurologist
Barriers to technology integration	
Too many apps	▪ “*because on my [Public Health Service] desktop I've got about 40 icons right. and each little, each little bit of [Public Health Service]'s got its own database and its own thing, like the emergency department's got its own software. The ICU has its own software. Mental health got its own software. I mean, each section has its own software. And they don't integrate actually, even even things like echocardiograms*” -Psychiatrist
Integration issues	▪ “*Something that could be integrated into the platform that's already there, of course would be easier. How, how difficult or how easy that is. I have no idea*” -Nurse Navigator ▪ “*The challenge. I think it would for the developers of the system would be to gain the permissions and the access for another piece of software to go into a health system, which is very protective. It's actually it's actually got a lot of different software programs. But it's quite protective about new ones coming in. And and the risks that they entail. Particularly privacy risks.*” - Psychiatrist
Internet issues in remote areas	▪ “*Internet. So, like, for example, like it's — it's ridiculous, but you can't always guarantee Internet connectivity*” -Neuropsychologist
Older people and technology	▪ “*a lot of that older patients aren't particularly tech savvy, so being able to access something like on a small screen on a mobile phone perhaps might be a bit too much for some of them*” -Neurologist
Stress caused by access of results	▪ “*you know, let – I'm talking like really worst-case scenario. But let's say a patient who has very severe anxiety saw the results and thought that's it, I've got dementia, you know, went off and harmed themselves because they thought, my life is over*” -Neuropsychologist
Should not increase clinician workload	▪ “*And as you say, the doubling up, we don't want to double up work for people either*” -Nurse Navigator ▪ “*yeah, my concern there is it's doubling up the work for the neuropsychologist to have to rewrite a different thing for a separate platform.*” -Neuropsychologist
Copyright issues with assessments	▪ “*Because you'll just have to think about, obviously, um, copyright issues*” -Neuropsychologist
Complexity of assessments	▪ “*it could result in some things being missed because ultimately, you know, there is a clinical observation and opinion that comes about that may not fit the algorithm.*” -Neuropsychologist
Other technical issues	▪ “*Timely, potentially more efficient as long as the program doesn't crash. We have had that happen with other programs. You think you could, okay? But you know, things just don't sign in and next thing you're like, “Where's the pen and paper? I need to go back to what works*” -Neuropsychologist

Beyond training support, participants suggested specific technological solutions to enhance the assessment process. The findings emphasised a need for technology that enabled neurologists to view neuropsychologist availability before making referrals, streamlining the referral process. The participants also highlighted the importance of developing technology capable of providing concise assessment summaries. Visualisations could be incorporated to improve interpretation, while search functionality would enable quick access to specific clinical information. Given these various requirements for managing both referrals and clinical information, strong support emerged for developing a centralised platform. This platform would not only manage referrals throughout the neuropsychological assessment process but gather necessary clinical information and facilitate sharing between various healthcare providers.

### Benefits and barriers to technology integration

Participants described the integration of technology within clinical pathways could provide numerous benefits (Table 2). The primary benefit is that technology could support the clinical processes, while reducing the risk of missed referrals. The technology could offer significant advantages to clinicians in remote or regional areas, where face-to-face services are limited. It would promote collaboration among stakeholders and enable monitoring of cognition over time, while providing patients access to their results.

Despite these benefits, several challenges were identified related to (1) technology, (2) user adoption and accessibility, (3) patient experience and concerns, and (4) assessment methodologies and constraints. Participants expressed concerns about the number of applications already in use within their workstations and preferred integrating the platform into existing systems. This would avoid the added burden of maintaining multiple systems. However, they acknowledged the integration challenges due to security and privacy concerns within healthcare infrastructure. Furthermore, given technology would benefit rural and remote regions, participants raised concerns about internet access in these areas (*Barrier 1*).

Challenges regarding the use of technology by people with PD and their support persons, particularly considering the older age of this population (*Barrier 2*) and the potential stress caused by sharing results before a feedback session (*Barrier 3*), were described. Additionally, the implemented technology needs to be easy to use for the physician, requiring minimal learning or training (*Barrier 2*).

Finally, several concerns emerged about conducting neuropsychological assessments online. Technical problems included program crashes during assessments, poor internet connectivity, and sign-in difficulties. These technical challenges often forced neuropsychologists to return to traditional methods. Copyright issues with assessment materials and challenges translating clinical observations used in neuropsychological assessment into an algorithm were reported as significant concerns (*Barrier 4*).

## Discussion

This research examined the complex landscape of neuropsychological assessments for people with PD across two healthcare service models. Through systematic analysis, we mapped a four-phase progression: the initial recognition of cognitive assessment needs, preliminary screening protocols, comprehensive assessment procedures, and post-feedback management. Our findings revealed variations between clinical pathways, particularly in their approach to healthcare delivery and stakeholder involvement. The study highlighted several challenges, including limited awareness, time constraints, long waiting periods for assessments, lengthy appointment times, and concerns regarding report length. The literature has also highlighted these challenges, which impact service access among people with PD and contribute to poor understanding of cognitive impairment and limited support and treatment (29).

According to the study finding, health technologies could support some of the healthcare processes. The implementation of technology in healthcare settings has been driven by goals of enhancing service efficiency, care quality, cost effectiveness, accessibility, and minimising wait times (30, 31). This broader movement toward technology integration in neuropsychology is exemplified by Singh et al. (32) hybrid neuropsychology model, which proposes developing technology-based practices, integrating data science, and collaborating with innovators from other field. Consequently, many health service improvement studies have adopted this approach when investigating technological interventions (33–37), including those focused on cognitive assessments (38, 39). Of these technologies, telemedicine for remote neuropsychological assessment was predominantly studied (40–42), as most studies demonstrated strong agreement between telehealth and face-to-face neuropsychological assessments (42). For instance, Carotenuto et al. (40) highlighted that Mini-Mental State Examination (MMSE) showed comparable results between traditional cognitive screening and telehealth administration.

This raises the question of which technological solutions could enhance these clinical pathways. Findings present design considerations for such technology. Integration emerged as a primary consideration, with stakeholders emphasising the need for solutions that seamlessly connect with existing Electronic Medical Records to avoid the burden of managing multiple platforms. This integration requirement becomes crucial given the complex nature of neuropsychological assessments and the need to maintain comprehensive patient records across different healthcare providers. Accessibility formed the second consideration, particularly given the diverse needs of remote and regional areas, as well as the varying technical proficiencies of different user groups. The study found that technology solutions must balance sophisticated functionality with user-friendly interfaces to accommodate both healthcare providers and individuals who may have limited technology experience. The third consideration centred on workflow support, emphasising streamlined referral management, summarised report presentation and improved result visualisation. This includes tools for scheduling, assessment tracking, simplified report generation, and lengthy reporting processes. A final consideration is the need for the technology to increase awareness of the role of cognitive assessment in PD.

However, the findings revealed several potential barriers to technology adoption, including security and privacy concerns, age-related challenges, usability issues, and copyright restrictions. Germine et al. (43) also identified major implementation barriers related to digital neuropsychology such as the impact on test interpretation and normative data, varying cognitive and motor demands across devices, and potential test obsolescence due to rapid technological advancement. These barriers can be identified and mitigated through appropriate design and development methodologies, as described by previous studies focusing on older populations (44) and technical-organisational implementation (45). Co-design represents one such methodology that has been recognised as important for conducting research with people who have PD and cognitive impairments (46, 47). This approach is particularly well-suited to PD populations, as recent research demonstrates that people with early to mid-stage PD maintain accurate awareness of their cognitive status and can reliably report on their difficulties (48), making them valuable partners in identifying authentic user needs. The co-design process involves actively involving people with lived experience into the design process, by treating them as individuals with equal creative input as designers in the development process (49). Several approaches can be implemented to facilitate involvement including the use of drawings, photographs and prototypes to explore and develop solutions (49). These co-design methodologies provide a pathway for developing technology solutions that are both technically robust and genuinely responsive to the complex needs identified in the clinical pathways.

### Strengths and limitations

Our study provides the first comprehensive mapping of neuropsychological assessment pathways for people with PD across two Australian public health services, incorporating perspectives from clinical experts. The use of journey mapping enabled detailed visualisation and analysis of complex clinical workflows. We achieved strong participation across various clinical roles, with fifteen participants representing different specialties and experience levels.

Several limitations should be noted. Firstly, our study focused on two public health services in Queensland, Australia, limiting generalisability to other healthcare settings or regions. Secondly, while we captured clinician perspectives, we did not include people with PD or their support persons, whose lived experience could provide valuable insights into the assessment process. Finally, we included clinical experts in the public health system, excluding perspectives from private practice and other healthcare providers who may have different experiences and approaches.

## Conclusion

This study provides insights into the current landscape of neuropsychological assessments for people with PD from the perspective of healthcare professionals across two public health services in Queensland, revealing a complex four-phase process with variations between clinical pathways. Our findings highlight challenges in the assessment process, including limited clinician awareness, time constraints, and lengthy waiting periods, while identifying opportunities for technology integration. The journey mapping approach successfully visualised these clinical pathways and identified areas for improvement through technological solutions. However, successful implementation of technologies must consider key barriers including system integration requirements, accessibility needs, and copyright restrictions for assessment tools. These findings suggest that while technology offers potential to enhance neuropsychological assessment, implementation must be designed to address barriers while maintaining assessment quality and accommodating the diverse needs of healthcare providers and patients. The PDCogniCare project will build upon this foundation to advance the development of technology-enabled neuropsychological assessments.

## Data Availability

The datasets presented in this article are not readily available because given the sample size it may be able to identify individuals participants, which is against what was approved in our ethics approval. Requests to access the datasets should be directed to Elton Lobo, elton.lobo@uq.edu.au.
